# Curcumin activates NLRC4, AIM2, and IFI16 inflammasomes and induces pyroptosis by up-regulated ISG3 transcript factor in acute myeloid leukemia cell lines

**DOI:** 10.1080/15384047.2022.2058862

**Published:** 2022-04-18

**Authors:** Yuru Zhou, Yunyuan Kong, Mei Jiang, Linju Kuang, Jinhua Wan, Shuyuan Liu, Qian Zhang, Kuai Yu, Na Li, Aiping Le, Zhanglin Zhang

**Affiliations:** a Departments of Blood Transfusion, The First Affiliated Hospital of Nanchang University, Nanchang, Jiangxi, China; b JiangXi Key Laboratory of Transfusion Medicine, Nanchang, Jiangxi, China; c Department of Physical Examination, The First Affiliated Hospital of Nanchang University, Nanchang, Jiangxi, China; d Departments of Clinical Laboratory, The First Affiliated Hospital of Nanchang University, Nanchang, Jiangxi, China; e Departments of Stomatology, The First Affiliated Hospital of Nanchang University, Nanchang, Jiangxi, China

**Keywords:** Curcumin, inflammasome, pyroptosis, leukemia, GSDMD

## Abstract

Curcumin, the primary bioactive component isolated from turmeric, has been found to possess a variety of biological functions, including anti-leukemia activity. However, the effect of curcumin in different leukemia cells vary. In this study, we demonstrated that curcumin induced the expression of AIM2, IFI16, and NLRC4 inflammasomes in leukemia cells U937 by increasing the expression levels of ISG3 transcription factor complex, which activated caspase 1, promoted cleavage of GSDMD, and induced pyroptosis. We also found that pyroptosis executor GSDMD was not expressed in two curcumin-insensitive cells HL60 and K562 cells. In addition, exogenous overexpression of GSDMD by lentiviral transduction in K562 cells increased the anti-cancer activity of curcumin, and inhibiting the expression of GSDMD by shRNA enhanced U937 cells to resist curcumin. The results showed that inducing pyroptosis is a novel mechanism underlying the anti-leukemia effects of curcumin.

## Introduction

Acute myeloid leukemia (AML) is a highly heterogeneous disease characterized by the accumulation of acquired genetic changes in myeloid progenitor cells that alter mechanisms of self-renewal, proliferation, and differentiation.^1,2^ AML could occur in any age group, especially in elder individuals. From 1990 to 2017, the incidence of AML gradually increased in the globe.^3^ The median overall survival (OS) after 5 years in adult AML patients is roughly 25% and around 10% in the patients above 60 years old.^2,4^ Hence, there is a high medical need to improve the outcome of AML patients.

Curcumin, an active ingredient derived from turmeric, has been recognized for its medicinal properties, including antioxidant, anti‐inflammation, radical‐scavenging, anti-solid and -blood tumor, and so on.^5–7^ The anticancer effects of curcumin mainly result from multiple biochemical mechanisms that are involved in the regulation of programmed cell death, such as apoptosis, autophagy.^5,7–9^ Recent studies have found that curcumin can induce pyroptosis of solid tumor cells.^10,11^ However, it is not clear whether pyroptosis is involved in the anti-leukemia effect of curcumin.

Pyroptosis is a rapid lytic cell death dependent on caspase-1/4/5/11 activity, which is activated by inflammasomes.^12,13^ Inflammasomes are multimolecular complexes containing pattern-recognition receptors (PRR), apoptosis-associated speck-like protein containing CARD (ASC), and effector caspases. Inflammasomes activate caspases and then cleave and activate specific members of pore-forming gasdermins. Cleavage gasdermin forms the pores in the plasma membrane, leading to the membrane defects and cytosolic protein release and induce programmed cell death. Recent studies found that the expression of some pyroptotic inflammasomes and gasdermins decreased in cancer cells.^14,15^ Drug-regulated pyroptosis promotes inflammatory cell death of cancer and inhibits proliferation and migration of cancer cells.^16,17^ Val-boroPro, the ‘inflammasome’ sensor protein CARD8 activator, which successively activates procaspase-1 to mediate pyroptosis in primary acute myeloid leukemia (AML) samples and most AML cell lines,^18^ suggesting that pyroptosis is suitable for the treatment of AML.

In this study, we found that curcumin induced the expression of NLRC4, AIM2, and IFI16 inflammasomes by upregulated ISG3 transcription factor complex in leukemia cell U937, which activates caspase 1 and promotes cleavage of GSDMD. In addition, we also found that the expression of GSDMD varied greatly in leukemia cell lines. GSDMD expressing cells were more sensitive to curcumin, while HL60 and K562 cells without GSDMD were not sensitive to curcumin. Our results demonstrated that pyroptosis may be a potential new mechanism of curcumin treating leukemia, and GSDMD is a biomarker to evaluate curcumin sensitivity in the leukemia therapy.

## Materials and methods

### Cells and reagents

Leukemia cell lines U937, K562, NB4, THP1, HL60, MV4-11, and Kasumi were maintained in RPMI-1640 containing 10% fetal bovine serum under 37°C with 5% CO_2_. HEK-293 T cells were maintained in DMEM containing 10% fetal bovine serum. Curcumin (Sigma, St. Louis, MO) was dissolved in DMSO as a stock solution at 5 mM. The antibodies against AIM2, NLRC4, and IFI16 were purchased from ABclonal Technology (Wuhan, China) Co.,Ltd. GSDMD antibody was from Proteintech Group, Inc(China). Annexin-V-FITC/PtdIns kit was purchased from Bestbio Biotechnology (Bestbio, China). Reverse transcription reagent and SuperReal qPCR PreMix (SYBR Green) reagent kit were purchased from TIANGEN Biotech (Beijing) Co., Ltd. The lentiviral expression vector pLVX-Puro and pLVX-shRNA1 vector and the lentiviral packaging plasmids were provided by Clontech Laboratories, Inc.

### GSDMD expression and shRNA plasmids construction, transfection, and lentiviral transduction

The vector pEGFP‑C1 was from Clontech Laboratories, Inc.B. The full-long GSDMD and N-GSDMD open reading frame (ORF) was cloned in pEGFP‑C1 to generate the pEGFP‑C1-GSDMD and pEGFP‑C1-N-GSDMD plasmid. The GSDMD was then inserted into the lentiviral vector pLVX-tetone-puro and transfected into 293 T cells together with the packing plasmids pSPAX2 and pMD2G at a ratio of 5:3:2 using EndoFectin™ (GeneCopoeia, Inc.) based on the manufacturer’s protocol. After transfection for 48 h, the virus was harvested and used to infect K562 cells. Stable clones expressing GSDMD (K562-GSDMD) and negative control (K562-TETONE) were then selected using puromycin. Stable cell clones transduced with the empty vector were used as the controls. The expression of GSDMD was measured after treating with 0.5 µg/ml doxycycline (cat. no. 24390–14; MedChemExpress).

Two construct encoding GSDMD-targeting shRNA sequence was designed (Sh1: gatcgtgtgtcaacctgtctatcaactcgagttgatagacaggttgacacacttttttg; Sh2: gatccagcacctcaatgaat -gtgtactcgagtacacattcattgaggtgctgttttttg) and synthesized. Constructs were cloned into the lentiviral vector pLVX-shRNA; Virus packaging and infection were seen above.

### RT-qPCR

Cells (2 × 10^6^) were grown in cell culture bottles and then treated with the indicated concentrations of drug for 24 h. Total RNA was extracted by TRIzol reagent (Invitrogen) and reverse-transcribed to the cDNA, and the following qPCR with the specific primers were as follows: AIM2: 5′-aacgtcttcaggaggagaag-3′ and antisense 5′-accataactggcaaacagcg-3′; IFTI16: sense 5′-tcagattgctgacttgatgg-3′, antisense 5′-tacctgacatttggccactg-3′; NLRC4: sense 5′-tgagagaacacatctgctgg-3′ and antisence 5′-accttctcgcagcaaatgatg-3′; GAPDH: sense 5′-tgacttcaacagcgacaccca-3′ and antisense 5′-caccctgttgctgtagccaaa-3′. The relative quantity of target gene expression was analyzed using the comparative CT (2-ΔΔCT) method.

### Cell counting kit 8 analysis (CCK-8)

100 µl of cell suspension (5000 cells/well) was seeded in 96-well plates and cultured for 0, 24, 48, and 72 h. To each well, 10 µl CCK-8 solution was added, and after 2 hours incubation, the absorbance at 450 nm was measured by a microplate reader (Multiskan Fc,Thermo). The relative growth of cells (OD(test)/OD(control)) was used to plot the growth curve.

### Western blot analysis

Proteins were extracted by 2x protein loading buffer (125 mM Tris-HCl (pH 6.8), 4% SDS, 5% 2-hydroxy-1-ethanethiol, 20% glycerol, 0.01% bromphenol blue). Cells (2 × 10^6^) were added with 100 µl loading buffer, boiled at 100°C for 5 minutes, then frozen for 5 minutes, repeated 3 times. Proteins from cell lysates (10 µl) were electrotransferred to nitrocellulose membranes after separated on 10% SDS-PAGE. Before being blotted with primary antibody overnight at 4°C, membranes were sealed for 1 h at room temperature in Tris-buffered saline-0.05% Tween-20 (TBST) containing 5% nonfat dry milk. After 3 × 10 min washes in PBS, membranes were incubated with peroxidase-conjugated secondary antibody for 1 hr. Following 3 additional 10- min washes with TBST, the proteins were imaged by enhanced chemiluminescence detection reagent and detected with Bio-Rad ChemiDoc XRS+ chemiluminescence imaging system (Bio-rad laboratories Inc.).

### Apoptosis analysis by FMC

10^6^ cells were harvested in the appropriate manner (centrifuged at 1,500 rpm for 5 min), 5 µl Annexin-V were added after adding 300 µl Annexin-V binding solution, and the mixture was placed at 25°C for 15 min. 10 µl PtdIns was added at 5 min, then the results were analyzed by cytometer (FC500, BACKMAN). Annexin V+ and/or PtdIns+ cells are apoptosis cells.

### Statistical analysis

All data were repeated three times and presented as mean ± SD (n = ≥3). SPSS13.0 software (SPSS Inc., Chicago, IL) was used for all the analyses. One-way ANOVA was used to analyze the gene expression after treated with different concentrations of curcumin. Two-way ANOVA was used to analyze the differences in proliferation by CCK-8 assay. Following ANOVA, Bonferroni’s post hoc test was used to determine significant differences. Student’s t-test was used to compare the apoptosis in experimental and control groups. All data are presented as mean ± standard deviation. P < .05 was considered to indicate a statistically significant difference.

## Results

### Curcumin induces the expression of AIM2, IFI16, and NLRC4 inflammasomes in leukemia cells U937

**Figure 1.**
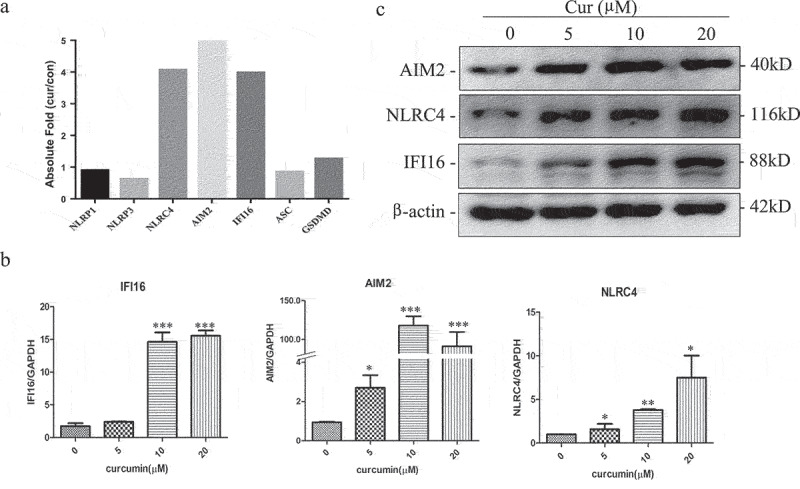
Curcumin induces the expression of AIM2, IFI16, and NLRC4 inflammasomes in leukemia cells U937.

Using the gene expression chip, we analyzed the changes of transcriptome in U937 cells after treated with curcumin. There were 861 genes that were upregulated and 866 downregulated more than twofold. Using the online Metascape enrichment analysis (https://metascape.org/) to identify different ontology sources of the change genes, we found that the upregulated genes are closely related to autophagy, M-phase arrest, apoptosis, and cytokine-mediated signaling pathways. The decreased genes are mainly related to Ribosome biological activity, protein translation, and MYC activation signal pathway (Figure S1). Interestingly, we found that AIM2, IFI16, and NLRC4, but not NLRP1 and NLRP3, which could form inflammasomes and induce pyroptosis, were significantly upregulated in the gene expression chip (Figure 1(a)).

(a) Cene expression chip analyzed the expression of NLRP1, NLRP3, NLRC4, AIM2, IFI16, ASC, and GSDMD in U937 treated with curcumin (10 μmol/L) for 24 h; (b) validation of NLRC4, AIM2, and IFI16 expression in U937 when treated with curcumin for 24 h by RT-PCR. Each value represents the mean ± SD (*P < .05, **P < .01, ***P < .001); (c) western blot array analyzed protein level of NLRC4, AIM2, and IFI16 h in U937 after treated with curcumin for 24 h.

In order to confirm the change of AIM2, IFI16, and NLRC4 in the gene expression chip, U937 cells were treated with different concentration of curcumin (0, 5, 10, and 20 µM), and RNA and protein level of AIM2, IFI16, and NLRC4 were determined by RT-qPCR and western blot, respectively. As shown in Figure 1(b,c), the mRNA and protein expression of the inflammasomes increased after treating with curcumin.

### Curcumin activated caspase-1 and promoted GSDMD cleavage in leukemia cells

**Figure 2.**
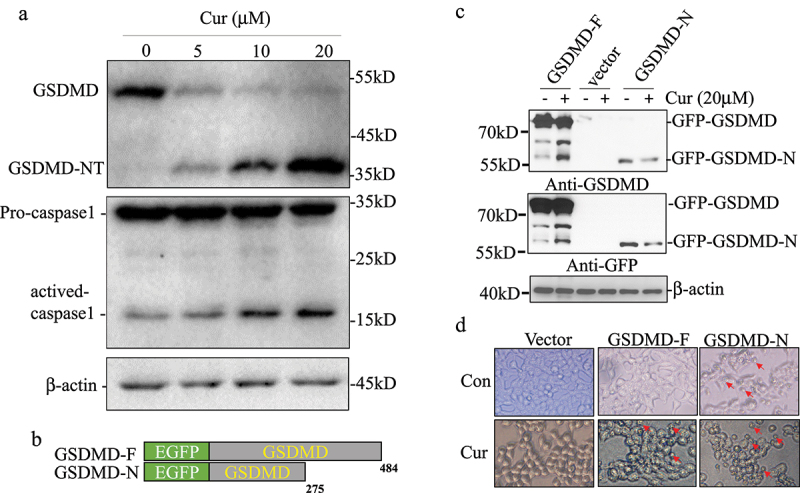
Curcumin activated caspase-1 and promoted GSDMD cleavage in leukemia cells.

Previous study reported that AIM2, IFI16, and NLRC4 inflammasomes can activate Caspase-1 and promote cleavage of GSDMD.^17^ Then, we evaluated whether curcumin induced cell pyroptotic death via activating Caspase-1 and GSDMD. We treated the U937 cells with different concentrations of curcumin (0, 5, 10, and 20 µM) and detected the cleavage of GSDMD and activation of caspase-1 by western blot. As seen in Figure 2(a), GSDMD decreased after treating with curcumin, and the cleavage GSDMD and activated caspase-1 increased in dose-dependent manners (Figure 2(a)).

(a) U937 cells treated with different concentration of curcumin (0, 5, 10, and 20 µM) and detected the cleavage of GSDMD and activation of caspase-1 by western blot. (b) The expression plasmids of GSDMD-F and GSDMD-N fused with EGFP were constructed. (c) HEK-293 T cells were transfected with GSDMD plasmids and analyzed the cleavage effect of GSDMD after treated with curcumin for 24 h by detecting with GFP and GSDMD antibody. (d) Expression of GSDMD-affected morphology of 293 T cells after treating with curcumin.

In order to confirm the curcumin-induced cleavage of GSDMD lead pyroptosis, we constructed the expression of plasmids GSDMD-F and pyroptosis executor GSDMD-N fused with EGFP and transfected in HEK-293 T cells for analyzing the cleavage effect of GSDMD by curcumin (Figure 2(b)). A cleavage GSDMD band with the same molecular weight as GSDMD-N was significantly increased after being treated with curcumin for 24 hours by detection with GFP and GSDMD antibody (Figure 2(c)). Compared with the control group, GSDMD had no effect on 293 T cell morphology, overexpression of GSDMD-N could promote cell swelling and death, and expression of GSDMD significantly promoted cell swelling and death after curcumin treatment (Figure 2(d)), suggesting that pyroptosis is involved in the antitumor effect of curcumin.

### Curcumin upregulated ISG3 transcription factor complex

**Figure 3.**
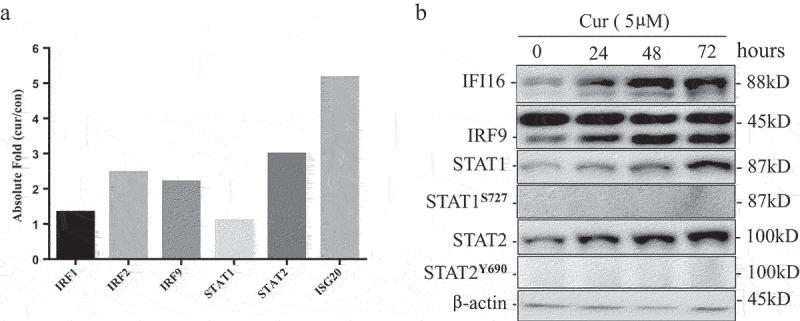
Curcumin up-regulated ISG3 transcription factor complex.

PHYIN family genes (IFI16 and AIM2) contain interferon response elements in the promoters, and their expression is regulated by IFN transcription factor complex.^19^ ISGF3 complex is composed of STAT1, STAT2, and IRF9. By microarray analysis, we found that the expression of STAT2 and IRF9 were increased, suggesting that curcumin may induce PHYIN family genes expression by activating ISGF3 (Figure 3(a)). To further test this hypothesis, we treated U937 cells with 5 µm curcumin and analyzed the protein expression and phosphorylation of ISGF3 complex by WB at 0, 24, 48, and 72 hours. The results in Figure 3(b) show that the protein levels of IFI16, STAT1, STAT2, and IRF9 increased in time-dependent manners. But we could not detect the phosphorylation of STAT1 and STAT2 after treating with curcumin. These results suggest that curcumin may promote the expression of interferon inducible PHYIN family (IFI16 and AIM2) by upregulating ISGF3 transcription factor.

(a) Gene expression chip analyzed the expression of IRF1, IRF2, IRF9 STAT1, and STAT2 in U937 treated with curcumin (10 μmol/L) for 24 h; (b) western blot array analyze protein level IRF9, STAT1, and STAT2 and phosphorylation of STAT1 and STAT2 in U937 after treated with curcumin for 24 h.

### Overexpression of GSDMD enhance anti-leukemia effect of curcumin

**Figure 4.**
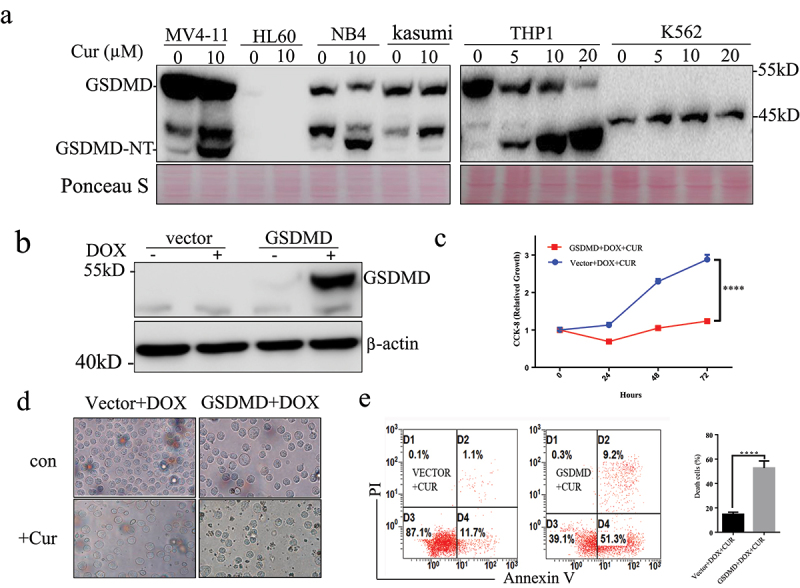
Overexpression of GSDMD enhance anti-leukemia effect of curcumin.

High expression of GSDMD has good prognosis in some solid tumors.^16^ Our previous studies have shown that different leukemia cells have different sensitivity to curcumin. We detected the expression of GSDMD in different leukemia cells. Curcumin could induce cleavage of GSDMD in some leukemia cell lines such as MV4-11, NB4, Kasumi, and THP1. Interestingly, K562 and HL60 cells, which were not sensitive to curcumin, did not express GSDMD (Figure 4(a)).

(a) Western blot array analyzed the expression of GSDMD in different AML cell lines and cleavage of GSDMD after treating with curcumin. (b) DOX induced expression of GSDMD in a stable GSDMD-K562 cell line. (c) CCK-8 array analyzed growth curves of the stable K562 cell lines after treated with curcumin at 24, 48, and 72 h. (d) Cell morphology changes was analyzed in the table K562 cell lines after treated with curcumin. (d) The apoptosis was analyzed in the table K562 cell lines after treated with curcumin.

To functionally investigate the role of GSDMD in anti-leukemia effect of curcumin, the present study established a stable GSDMD-K562 cell line that overexpressed GSDMD following DOX exposure. As presented in Figure 4(b), the GSDMD protein was detected after 24 h of DOX exposure (0.5 µg/ml) but not in the vector control cell lines. The effect of GSDMD overexpression on the proliferation of K562 cells after treatment with curcumin was then measured. Following exposure to 0.5 µg/ml DOX, proliferation of K562-GSDMD cells was significantly reduced compared with K562-TETONE cells after treatment with DOX (P < .0001) (Figure 4(c)). Cell morphology and apoptosis analysis demonstrated that GSDMD overexpression promoted death of K562 cells after treatment with curcumin (Figure 4(d,e)).

### Knock-down GSDMD inhibits curcumin-induced death of U937 cells

**Figure 5.**
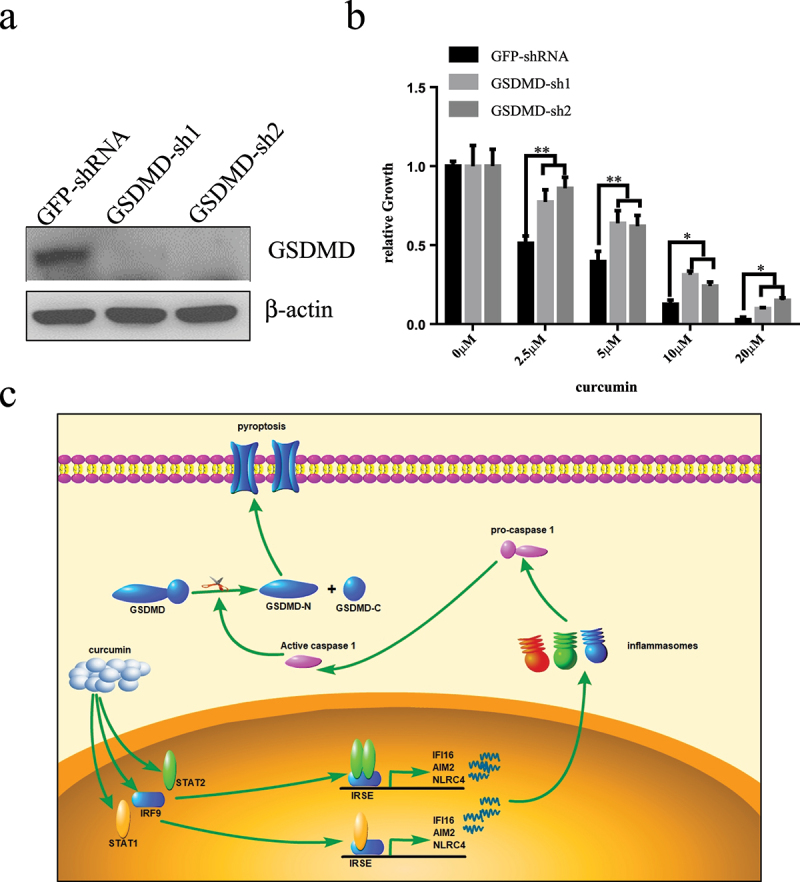
Knock-down GSDMD inhibits curcumin-induced death of U937 cells.

To further explore the role of GSDMD in curcumin anti-leukemia, we used RNA interference technology to inhibit the expression of GSDMD in U937 cells and analyzed whether it could change the sensitivity of U937 cells to curcumin. U937 cells were infected with two GSDMD-specific shRNA virus, and both shRNAs could significantly inhibit the expression of GSDMD (Figure 5(a)). We analyzed the effect of GSDMD knockdown on inhibiting cell proliferation by curcumin. Cells were treated with curcumin (0, 2.5, 5, 10, and 20 µM) for 24 hours, and proliferation was analyzed by CCK-8 assay. The results in Figure 5(b) show that the curcumin could inhibit the growth of U937 cells with dose-dependent manners in both control and GSDMD-knockdown group. Compared with the control group, proliferation inhibition of GSDMD-knockdown group is significantly lower at the same concentration of curcumin, indicating GSDMD could enhance anti-leukemia effect of curcumin.

(a) GSDMD-specific shRNA or nonspecific (GFP) shRNA lentivirals infected U937 cells for 120 h, and the knockdown efficiency of endogenous GSDMD was analyzed by western blot. (b) CCK-8 array analyzed affection of knocking-down GSDMD on the growth inhibition of U937 cells after treating with curcumin for 24 h. (c) Schema of the mechanism for curcumin induces pyroptosis in AML cells.

## Discussion

Prior studies have shown that curcumin exert anti-cancer effects by induction of apoptosis and autophagy; however, we demonstrate that curcumin induces cell death by pyroptosis in AML cells. Pyroptosis, triggered by various pathological stimuli, such as stroke, heart attack, or cancer, is cellular inflammatory.^12,13,20^ Although a large number of studies have found that curcumin has anti-inflammatory effects, there are also evidences that curcumin and its derivatives can induce pyroptosis. Jill M. Miller et al.^11^ found that curcumin treatment in human malignant mesothelioma cells resulted in pyroptosis via activating caspase-1 induced by NLRP3 inflammasome. Liping Chen et al.^21^ found that the curcumin derivative exhibited good anti-tumor activity both in vitro and in vivo via the switch of apoptosis-to-pyroptosis. Wan-Feng Liang^10^ found that curcumin activates ROS signaling to promote pyroptosis in hepatocellular carcinoma HepG2 cells. In our studies, we found that heterologously overexpressing GSDMD enhanced sensitivity of K562 cell to curcumin, and inhibition of GSDMD with shRNA in U937 cells enhance curcumin anti-leukemia effect, indicating that the sensitivity of leukemia cells to curcumin is clearly related to pyroptosis.

Classic pyroptosis needs activating caspase 1 by inflammasomes.^22^ Assembly of the inflammasome complex is initiated by nucleotide-binding domain, and leucine-rich repeat receptors (NLRs) are absent in melanoma 2 (AIM2)-like receptors (ALRs).^23^ So far, 22 NLRs and 4 ALRs have been identified in human. Since the ability of NLRP1 to form an inflammasome complex was described in 2002, it is now clear that other members of the NLR and ALR family, including NLRP3, NLRC4, and AIM2, can also assemble the inflammasome.^24^ Emerging evidence indicate that human NLRP2, NLRP7, IFI16, and Pyrin also activate caspase 1.^25^ In leukemia cells, we found that curcumin promote the expressions of AIM2, IFI16, and NLRC4 but not NLRP1 and NLRP3. Studies have shown that the expressions of NLRC4, AIM2, and IFI16 genes are regulated by interferon.^26^ Classical interferon signaling pathway is well characterized to engage a cascade of signaling events to phosphorylate STAT1 and STAT2, which trigger the formation of ISGF3 containing STAT2, STAT1, and the IRF9 and promote the transcription of IFN-induced genes (ISGs). Besides, heteromers formed by STAT2 and STAT1 or IRF9 without phosphorylation also induce expression of ISGs through non-canonical pathways.^27^ In our study, we found that STAT1, STAT2, and IRF9 increase after treated with curcumin in leukemia, but we could not detect the phosphorylation of STAT1 and STAT2, indicating that curcumin promoted the expressions of AIM2, IFI16, and NLRC4 through non-canonical signal.

GSDMD, which is mainly expressed in the gastrointestinal tract and skin, is a 53-kDa protein located downstream of the pyroptotic caspases.^13^ As mentioned previously, GSDMD is an executioner of pyroptosis, which can be cleaved by pyroptotic caspases and form the cellular membrane pores. In response to the stimulation, the GSDMD N-terminal domain can bind to phosphatidylinositol phosphates of the cell membrane. The binding could be further enhanced by the interaction of GSDMD N-terminal domain, phosphatidic acid, and phosphatidylserine and resulted in pore formation, cellular osmotic pressure change, cell membrane lysis, and pyroptosis. There are some studies indicating that certain drugs or molecules could trigger GSDMD-mediated pyroptosis in various types of cancer.^16,17,28^ In our studies, we analyzed the expression of GSDMD in different leukemia cells, and we found that GSDMD expression was not detected in curcumin-insensitive K562 and HL60 cells, while it was high in curcumin-sensitive U937 and other leukemia cell lines. It was also found that curcumin could promote the expression of GSDMD in a variety of leukemia cells. These data indicate that GSDMD can be used as a biomarker to evaluate curcumin sensitivity in the leukemia therapy.

In summary, here we have discovered that curcumin can induce leukemia cell death by increasing apoptosis and pyroptosis and that activated AIM2, IFI16, and NLRC4 inflammasomes play a key role in this process. In addition, the anti-leukemia effect of curcumin is affected by the expression of GSDMD. Thus, pyroptosis may be a potential new strategy for treating leukemia, and GSDMD is a biomarker to evaluate curcumin sensitivity in the leukemia therapy.
